# Group Differences in the Psychological Integration Path of the Rural-to-Urban Migrants: A Conditional Process Analysis

**DOI:** 10.3390/ijerph182111463

**Published:** 2021-10-31

**Authors:** Liu Yang, Qinyao Wu

**Affiliations:** 1 Institute of Local Governance, Yangtze Normal University, Chongqing 408100, China; liu.yang@yznu.edu.cn; 2 School of Communication, Yangtze Normal University, Chongqing 408100, China

**Keywords:** migrant workers, psychological integration path, conditional process analysis, group differences, perceived discrimination

## Abstract

At present, income and welfare inequality between migrant workers and urban natives has improved in China, but there are still many “semi-urbanized” migrant workers, whose psychological integration into the migrant city is very important for their mental health. By using a second stage conditional process model to decompose the effect of income on psychological integration into direct and indirect effects, this study explores the different psychological integration paths of migrant workers in different migration ranges, based on the data of the China Migrants Dynamic Survey (CMDS). The results show that the total effect of income on psychological integration is positive, and the value of inter-provincial samples is twice that of full samples. There is a significant difference in psychological integration paths between inter-provincial and intra-provincial samples, and when social comparison acts as a mediator, income has no direct effect on psychological integration of intra-provincial samples, while the direct and indirect effects of inter-provincial samples account for half of each other. Perceived discrimination played a reversed moderated role in the effect of social comparison on psychological integration, i.e., the lower the degree of perceived discrimination, the stronger the positive effect of social comparison on psychological integration, and vice versa. Therefore, according to the actual needs of different groups, relevant policies should be gradually adjusted to improve the psychological integration of migrant workers, thus contributing to their mental health.

## 1. Introduction

With the development of industrialization and urbanization, the expansion of urban boundaries and the cross-regional migration of population have become a normal status. By the end of 2020, there were 285.6 million migrant workers in China, of which 169.59 million had left the local area [1]. They provide abundant labor resources for cities and promote the development of the urban economy. However, there are some significant differences in social welfare between migrant workers and local residents, which hinder the better integration of migrant workers into cities [2]. Meanwhile, the internal differentiation of migrant workers leads to different psychological adaptation processes of different groups [3].

Social integration is a multi-dimensional progressive concept [4]. Different scholars divide social integration into different dimensions, but generally speaking, it includes economic, social, cultural, and psychological dimensions [5,6]. The progressive process of dimensions usually starts with the integration of economic dimension and finally deepens to the psychological dimension. Therefore, psychological integration is not only a dimension of social integration but also the target and result of social integration [7]. The psychological integration process of migrant workers is essentially the transmission of social integration from the economic dimension to the psychological dimension. How exactly is this process carried out? What roles do factors of different dimensions play in this transmission process? Are there any significant differences in the psychological integration paths between different groups of migration range? For these problems, the existing studies involve less and lack systematic empirical conclusions. Therefore, based on the data of Special Survey on Social Integration and Mental Health of China Migrants Dynamic Survey in 2014, this article systematically deconstructs the effects of economic, social, and cultural factors on the process of psychological integration of migrant workers by using a moderated mediation model and tries to explore the different psychological integration paths of migrant groups in different migration ranges.

## 2. Theoretical Model and Hypotheses

### 2.1. Theoretical Model

Urbanization has been advancing at an unprecedented speed in the history of the world and has become an important phenomenon and dimension in the development of economic and social modernization in various countries. The early theories or models that explained the migration mechanism of population mainly include the dual sector model [8], the push–pull theory [9], the human capital theory [10], the Harris–Todaro model [11], etc.

According to the general law of development, the urbanization process is not only the centripetal force of population and resources flowing to cities but also the centrifugal force of outward expansion. Since the end of World War II, in the social development of the United States and other countries, the phenomenon of population returning from big cities and megacities to small cities and even rural areas has appeared. This process is called counter-urbanization. Berry believes that the United States in the 1970s was a turning point, and the essence of the counter-urbanization process is the decline of scale, density, and heterogeneity, which is a movement that changes the population distribution from a relatively concentrated state to a less concentrated state [12]. According to his view, “counter-urbanization” and “de-urbanization” have led to the disintegration of American cities, and the population distribution pattern has changed from urbanization to counter-urbanization. Cloke explains counter-urbanization from the perspective of the countryside and thinks counter-urbanization is rural revival [13]. Rural gentrification is seen as a manifestation of counter-urbanization or “comfortable migration” [14,15].

The definition of psychological integration has developed from a one-way identity of “giving up all other aspects of one’s identity and gradually thinking of oneself as a member of the immigrant community” [16] to a two-way identity; it contains the contents of identification and acceptance, which are embodied in the identification of individuals with society and the acceptance of individuals by society [17,18]. Some scholars define psychological integration as a process of change in terms of psychological and emotional identification with one’s own social identity and belonging, as well as the social distance between oneself and local residents [19]. That is, social integration can only be truly achieved if individuals have a sense of identity and belonging to the place where they have migrated [20,21]. Therefore, the process of psychological integration goes from economic, social, and cultural factors to psychological factors, and this process usually starts with economic factors.

Income is a major indicator of the economic dimension of social integration. Data from China’s previous population censuses and National Dynamic Monitoring Survey of Migration all show that cities in relatively developed areas are places of concentrated migration. This is mainly due to higher income, more employment opportunities, and well-developed service industries in these regions [22]. At present, there is no consensus among scholars on how income affects the process of social integration. Most studies have shown that income is a positive factor affecting social integration, but some scholars have found that income has no significant impact on it [23], and there was even a periodic phenomenon of the “income paradox” [24]. Therefore, the impact of income factors on different dimensions of social integration should be different, and the existence of social comparison is one of the important reasons for this difference. Social comparison is the process of comparing one’s situation and status with that of others [25].

Social comparison theory was put forward by Leon Festinger, which means that each individual makes self-evaluation by using others as the yardstick of comparison in the absence of objectivity [26]. Talking about the mediating role of social comparison, a study found that upward social comparison mediated the relationship between SNS usage and users’ subjective well-being [27]. Another study revealed that emotions based on social comparison play a mediating role between the social comparison orientation and psychological well-being of Facebook users [28].

Discrimination has been identified as a major stressor and impact on migrant health [29]. Migrants usually reported a higher level of perceived discrimination [30]. Efforts aiming at reducing discrimination and enhancing integration for migrants help with protecting migrants’ health [31]. The concept of social integration also originated from the study of social exclusion. Current studies focus more on the ability and willingness of migrant workers to integrate, as well as some “hard barriers” in terms of institutional obstacles, and less on the acceptance attitude and willingness of local residents [32]. In fact, the “soft barriers” formed by the discrimination and exclusion of local residents in the cities where they migrated are also important obstacles to the social integration of migrant workers. Especially for the psychological integration, when individuals feel excluded or discriminated against in the social environment for a long time, and lack of effective coping methods and supporting resources, social alienation is likely to be triggered [33,34]. Social alienation is not only closely related to criminal behaviors, abnormal peer relationships, and other social risk signals but also has a direct negative impact on the physical or mental health of individuals [35,36]. In recent years, the perception and reaction of vulnerable groups to discrimination has become the focus of social exclusion research [37]. Based on this, we speculate that the perceived level of external discrimination of migrant workers will affect the transmission of social integration from the economic dimension to the psychological dimension.

Due to the vast territory of China, geographical separation is an important factor for the cultural differences among different regions [38]. From the cultural dimension, it is more difficult for migrant workers of inter-provincial migration to integrate into the local society than for those of intra-provincial migration. Therefore, whether the range of migration is cross-provincial is an important factor in the cultural dimensions of social integration. By analyzing whether there is a significant difference in psychological transmission paths between inter-provincial and intra-provincial migrant workers, we will examine the influence of cultural dimension factors on the process of psychological integration.

**Figure 1:**
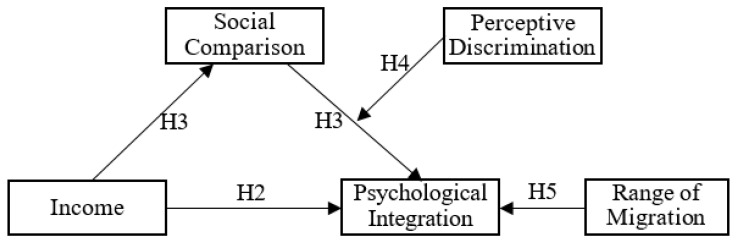
The theoretical hypothesis model of psychological integration paths.

In view of the above, we constructed a theoretical model of psychological integration paths, as shown in Figure 1. This model framed the main hypotheses in this study, which are introduced in Section 2.2. Hypotheses.

### 2.2. Hypotheses

For a concise and clear description, the direct influence of income on psychological integration is defined as direct effect, the influence of income on psychological integration through social comparison is defined as indirect effect, and the overall influence of income on psychological integration is defined as total effect, then: total effect = direct effect + indirect effect. The level of subjective self-assessment formed by comparison with surrounding people is defined as social comparison, and the degree of discrimination that one feels is defined as perceived discrimination. The income mentioned in the article refers to nominal income. Thus, we propose five hypotheses to be tested.

**Hypothesis** **1** **(H1).** *The total effect of income on psychological integration is positively significant.*

**Hypothesis** **2** **(H2).** *When there is a social comparison, the direct effect of income on psychological integration is significant.*

**Hypothesis** **3** **(H3).** *The indirect effect of income on psychological integration through social comparison is significant, that is, social comparison plays a mediating role in the process of income affecting psychological integration.*

**Hypothesis** **4** **(H4).** *The perceived discrimination has a moderating effect on the process of social comparison affecting psychological integration, that is, the level of perceived discrimination will affect the size of the indirect effect.*

**Hypothesis** **5** **(H5).** *In the above four hypotheses, there are significant differences between the estimated results using the inter-provincial samples and those using the intra-provincial sample, that is, the psychological integration path of the inter-provincial migrant workers is different from that of the intra-provincial ones.*

## 3. Materials and Methods

### 3.1. Data and Sample

**Figure 2:**
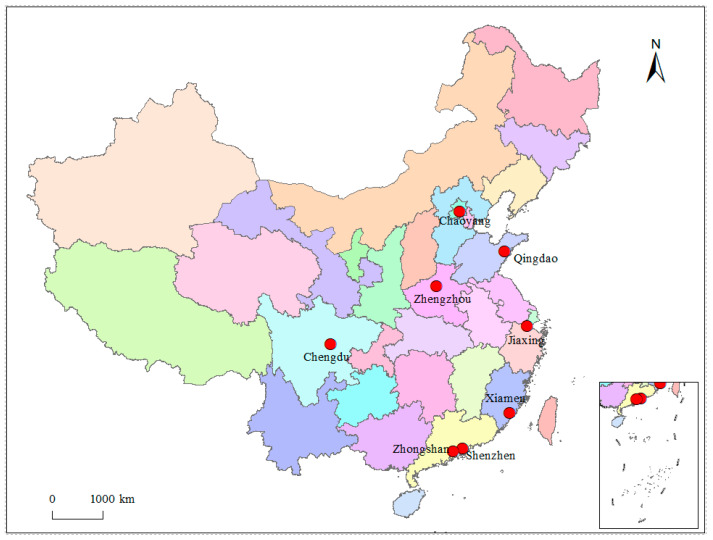
Location map of the sampling cities.

This study uses the data of Special Survey on Social Integration and Mental Health of China Migrants Dynamic Survey (CMDS) 2014 to make an empirical analysis. As shown in Figure 2, the survey contained eight cities (or districts of municipalities directly under the central government): Chaoyang in Beijing, Qingdao in Shandong, Xiamen in Fujian, Jiaxing in Zhejiang Province, Shenzhen in Guangdong, Zhongshan in Guangdong, Zhengzhou in Henan, and Chengdu in Sichuan, covering the eastern, central, and western regions and representing different urban locations, population scales, economic levels, and industrial types. The objects of the survey were non-local hukou residents aged 15–59 years old who had lived locally for one month or more as of May 2014. Multi-stage stratified random sampling was conducted in eight cities (districts). The total number of effective samples in eight cities was 15,997, which basically avoided the problem of homogeneity of samples. As the object of this study is migrant workers, the rural hukou samples were selected, and the final valid migrant worker samples totaled 13,925.

### 3.2. Variables

#### 3.2.1. Dependent Variable

The research focuses on the psychological transmission mechanism of social integration, so the results of psychological integration are taken as the object, defined as psychological belonging (*Bel*), and set as the dependent variable. The measurement of psychological belonging is influenced by many uncertain factors, but it is a relatively subjective judgment, which can be reflected by the self-evaluation of the interviewees. There are three related items in the social integration scale of the questionnaire, which are “feeling of belonging to the city”, “feeling of being a member of the city”, and “thinking of oneself as part of the city”. The measurement scale is a Likert scale. Participants selected items based on their feelings and assigned the following values: 1 = completely disagree, 2 = disagree, 3 = basically agree, and 4 = completely agree.

The data corresponding to these three items were found to be highly collinear by a correlation test. Therefore, an integer *K* with a value range of 3–12 was obtained by summing up the scores of the three items, which were assigned according to the value range of *K* and used as the substitute variable of psychological belonging (*Bel*). The assignment rules are as follows: when *K* ≤ 3, *Bel* = 1; when 3 < *K* ≤ 6, *Bel* = 2; when 6 < *K* ≤ 9, *Bel* = 3; when 9 < *K* ≤ 12, *Bel* = 4. The higher the value of *Bel*, the stronger the sense of psychological belonging.

#### 3.2.2. Independent Variable

Since the study of the psychological transmission mechanism of social integration is mainly based on the decomposition of the effect of income on the psychological integration of migrant workers, the factors closely related to the theoretical hypothesis are taken into account in the selection of the independent variables.

Income (*Inc*), as the main indicator variable of economic dimension, is the starting point of the psychological integration process, and it is set as the core independent variable. The relevant items in the questionnaire included “personal income in the last month or last employment month” and “average monthly income of family in local”. The formation of psychological feelings cannot be separated from the family background, and there is no significant difference between the estimation results using “personal income in the last month or last employment month” or “average monthly income of family in local”. Therefore, in the formal model, the data of “average monthly income of family in local” after logarithmic processing is used as a substitution variable of income level (*Inc*).

#### 3.2.3. Mediating Variable

In order to examine whether there is an indirect (mediating) effect of income on psychological belonging through social comparison (*Comp*), three items of social comparison were selected from the questionnaire: “compare your income and occupation with that of your relatives, friends, and colleagues in your hometown”, “with that of your relatives, friends, and colleagues in your current residence”, and “with that of people in whole society”, then “what level you think you are in”. The measurement scales were all 10-level Likert scales, and the higher the value from 1 to 10, the higher the interviewees’ subjective assessment of their socioeconomic status through social comparison. After testing, the data of these three items are highly collinear, so we continue to use the same processing method as the psychological belonging to assign values, and the specific process will not be repeated.

#### 3.2.4. Moderating Variable

To verify the moderating effect of perceived discrimination (*Desp*) on indirect effect, the score value of “I feel that local people look down on me” in the questionnaire was used as a substitute variable for perceived discrimination. The measurement scale is a Likert scale, with values from point 1 to 4, where the higher score means the higher degree of perceived discrimination. 

#### 3.2.5. Grouping Variable

In order to test whether the path of psychological integration of migrant workers in inter-provincial migration was different from that of ones in inter-provincial migration, a grouping variable was set up, which represents the range of migration (*Range*). The grouping variable Range entered the model as a dummy variable, where the value of inter-provincial migration sample is “1”, and the value of intra-provincial migration sample is “0”.

#### 3.2.6. Control Variable

Based on the existing studies and the availability of data, to control for other factors that may affect the mediating variable and dependent variable, we screened out other control variables, including gender, age, level of education, time of current migration, self-assessment of physical health, working hours per week, and whether or not they accepted free training provided by government.

The descriptive statistics for each variable are shown in Table 1.

### 3.3. Modeling

In order to decompose the direct and indirect effects of income on psychological integration, i.e., to verify the mediating role of social comparison and the moderating effect of perceived discrimination, we draw on the modeling method of Hayes [39,40], constructing a second stage conditional process model based on the theoretical hypothesis model.

First, without considering the range of migration, a full-sample model (Model 1) is obtained by using Equations (1)–(3).

(1) $$Comp=i_{a}+a_{0}Inc+a_{k}U_{k}+\mu_{a}$$

(2) $$Bel=j_{b}+b_{0}Inc+b_{1}Comp+b_{2}Desp+b_{3}Comp\times Desp+b_{k}U_{k}+\mu_{b}$$

(3) $$Bel=i_{c}+c_{0}Inc+c_{k}U_{k}+\mu_{c}$$

where, *U* is a set of control variables and enters the model as a covariate, and the value range of *k* is an integer from 4 to 10. Equation (1) represents the path regression equation of the independent variable (*Inc*) and control variable (*U*) to the mediating variable (*Comp*); Equation (2) represents the path regression equation of the independent variable (*Inc*), control variable (*U*), mediating variable (*Comp*), moderating variable (*Desp*), and interaction term of mediating variable and moderating variable (*Comp* × *Desp*) to the dependent variable (Bel); Equation (3) represents the total regression equation of the independent variable (*Inc*) and the control variable (*U*) to the dependent variable (*Bel*).

Then, the grouping variable (*Range*) was substituted into the model in the form of a dummy variable, and the Equations (4)–(6) were used for modeling to obtain the inter-provincial migration model (Model 2).

(4) $$Comp=j_{d}+d_{1}Inc+d_{k}U_{k}+d_{11}Range+\mu_{d}$$

(5) $$Bel=j_{e}+e_{0}Inc+e_{1}Comp+e_{2}Desp+e_{3}Comp\times Desp+e_{11}Range+e_{k}U_{k}+\mu_{e}$$

(6) $$Bel=i_{f}+f_{0}Inc+f_{k}U_{k}+f_{11}Range+\mu_{f}$$

Similarly, Equations (4) and (5) are path regression equations for the inter-provincial migration model, and Equation (6) is the total regression equation for it.

## 4. Results

In order to examine whether there is a mediating effect of social comparison and a moderating effect of discrimination perception in the process of income affecting psychological integration, we used the program PROCESS 3.5 to analyze the models. A bias-corrected confidence interval (CI) was constructed using the non-parametric Bootstrap method to estimate the conditional indirect effect of the model, that is, the mediating effect with moderating effect. The criterion is to observe whether zero is included between the upper and lower bounds of CI. Table 2 shows the estimated results of the main variables in Equations (1)–(6).

### 4.1. Total Effect of Income

The total effect of income on psychological integration can be tested by the total regression equation. The column “total effect” in Table 2 shows the estimation results of the total regression equations for Model 1 and Model 2, when controlling for other factors. The results show that income has a significant positive total effect on psychological belonging, and the total effect value (0.047) of inter-provincial migration model is about twice that of the full-sample model (0.022), indicating that total effect of income of inter-provincial migrant worker samples on psychological integration is twice that of the full samples.

This confirms well that the inter-provincial income gap is one of the main reasons for the inter-provincial migration of the labor force, which is consistent with the conclusions of many previous studies. Thus, Hypothesis 1 is accepted, and the part of Hypothesis 5 about Hypothesis 1 is also accepted.

### 4.2. Mediating Role of Social Comparison

The effect of income on psychological belonging through social comparison can be tested by a path regression equation. The columns “direct effect” and “conditional indirect effect” in Table 2 show the estimation results. After eliminating the influence of control variables, both Model 1 and Model 2 have significant conditional indirect effects at different levels of moderating variable. This means the mediating effect of social comparison existed, indicating that income has an indirect effect on psychological belonging through social comparison. The values of conditional indirect effect in two models are very close (Models 1: 0.023, 0.019, and 0.015; Model 2: 0.022, 0.019, and 0.016), which means that there is no significant difference in statistics. 

The direct effect of Model 1 is zero and is not statistically significant, indicating that the impact of income on psychological belonging has only an indirect effect, without having a direct effect. The direct effect of Model 2 is significant, and the estimated value of direct effect and conditional indirect effect is about 0.02, which indicates that the effect of income on psychological belonging is half through the direct effect of income and the other half through the indirect effect of social comparison. 

The results indicate that the psychological integration path of inter-provincial migrant workers is significantly different from that of intra-province ones and further confirms that the income gap between different provinces is an important reason the inter-provincial migration of labor force. Thus, Hypothesis 2 is not accepted in Model 1, it is accepted in Model 2, and the part of Hypothesis 5 about Hypothesis 2 is also accepted. Moreover, Hypothesis 3 is accepted in both models, but the part of Hypothesis 5 about Hypothesis 3 is not accepted.

### 4.3. Moderating Effect of Perceived Discrimination

The moderating effect of perceived discrimination on the stage of social comparison affecting psychological belonging (the second stage of indirect effect in mediating model) can still be examined by path regression equations; Table 3 presents the complete estimated results of four path equations for two models. The results show that in Model 1 and Model 2, the interaction coefficients of social comparison and perceived discrimination are both negative and statistically significant, indicating that perceived discrimination plays a moderating role in the stage of social comparison affecting psychological integration. In other words, the lower the degree of perceived discrimination, the stronger the positive effect of social comparison on psychological belonging, i.e., the stronger the indirect effect; the higher the degree of perceived discrimination, the weaker the positive effect of social comparison on psychological belonging, i.e., the weaker the indirect effect.

This conclusion can also be shown by the magnitude of conditional indirect effect when the moderating variable is at different levels in Table 2, which also indicates that the reverse moderating effect of perceived discrimination is significant. When the level of perceived discrimination is low, the value of conditional indirect effect is high, but when the level of perceived discrimination is high, the value of conditional indirect effect is low. The coefficients of the interaction terms in full-sample model and inter-provincial migration model are very close, being −0.011 and −0.010, respectively, which shows that there is no significant difference in the moderating effect of perceived discrimination between two models. Thus, Hypothesis 4 is accepted, but the part of Hypothesis 5 about Hypothesis 4 is not accepted.

**Figure 3:**
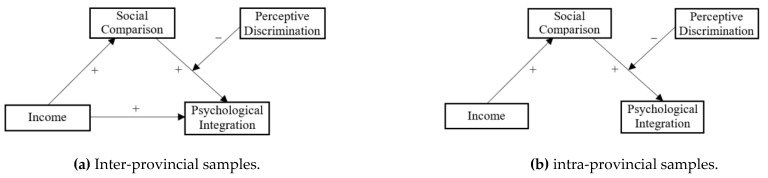
Psychological integration paths of migrant workers in different groups.

Figure 3 shows the test results of the theoretical hypothesis model (Figure 1). There are significant differences in psychological integration paths between groups with different ranges of migration.

## 5. Discussion

Based on the results of this study, the following suggestions are put forward to substantially promote the psychological integration of migrant works in China. 

First, the policy of equal treatment for migrant workers and local residents should be promoted and implemented as soon as possible. The analysis of psychological integration of migrant workers shows that social comparison plays a partial or even complete mediating role in this process, and the subjective self-evaluation grade of migrant workers’ social and economic status formed through social comparison has a significant positive effect on their psychological integration. Therefore, in deepening the reform of the hukou system (household registration system in China), it is imperative to promote and implement policies and measures for the equalization of public service at the same time, such as rationally allocating resources in basic education, medical care, housing, and other aspects and providing more urgent public services that match the actual needs of migrant workers so as to improve the efficiency of public service supply. This not only promotes social fairness but also improves the social comparative subjective self-evaluation level of migrant workers, thus promoting their psychological integration.

Second, an inclusive neighborhood environment should be created. It can give full play to the role of ties between neighbors and make migrant workers integrate into the local life. The study on the moderating effect of perceived discrimination shows that the lower the degree of perceived discrimination, the stronger the positive effect of social comparison on psychological integration. Therefore, while striving to provide more public services to meet the actual needs of migrant workers, we should also strengthen social recognition, social acceptance, and psychological counseling. In practice, it is necessary to integrate various neighborhood resources and give full play to the neighborhood management services as a link for migrant workers to integrate into local life. Neighborhoods with more migrant workers should further enrich and improve their cultural life, promote their participation in neighborhood activities, participate even more in the management, and strengthen the communications and interactions between local residents and migrant workers. By creating a neighborhood atmosphere conducive to eliminating discrimination and exclusion by local residents, migrant workers should receive guidance and assistance to better integrate into local life. On the one hand, it can make migrant workers feel more accepted and respected, reduce their perception of discrimination, and improve their psychological integration. On the other hand, it is also helpful to eliminate the harm of negative emotions to the physical and mental health of migrant workers and maintain the stable development of a harmonious society.

Third, cultural education and vocational training provided for inter-provincial migrant workers should be guided by demand matching. Through group comparison, this study found that income level directly affects the psychological integration of inter-provincial migrant groups, and the increase of income can significantly enhance their sense of psychological belonging. As a result, cultural education and vocational training for inter-provincial migrant workers should be strengthened in a targeted way, such as seminars on local language and culture and vocational training that is more appropriate to the local industrial structure. These measures will effectively improve the local language and cultural adaptation ability and vocational skills matching degree of inter-provincial migrant groups. This not only enables them to obtain more local employment opportunities but also increases the possibility of reasonably obtaining higher income, thus more effectively promoting the economic, social, cultural, and even psychological integration level of the inter-provincial migrant groups.

However, there were limitations to this study. First of all, as a cross-sectional study, the conclusions of comparing the differences of migrant workers’ psychological integration paths in different migration ranges is time-sensitive, so our future research needs the support of longitudinal data. In addition, the object of this study only focuses on migrant workers living in cities, but with the rapid development of urbanization in China, factors affecting immigration decision-making become more complex, and the phenomenon of counter-urbanization or gentrification cannot be ignored. Many scholars in Europe or America have conducted in-depth research on counter-urbanization and gentrification [12,13,14,15,41,42,43,44,45,46,47,48,49,50]. Therefore, in our future surveys and studies, we will focus on other types of migrants.

## 6. Conclusions

This study explored the differences in the psychological integration paths of migrant workers between the different migration range groups and verified the mediating role of social comparison and the moderating effect of perceived discrimination in the process of income influencing psychological integration. 

The findings are as follows. (1) The total effect of income on psychological integration is positive and significant, and the total effect on inter-provincial migrant worker samples is twice that of the full samples. (2) The psychological integration path of inter-provincial migrant workers is significantly different from that of intra-provincial ones. The effect of income on psychological integration of intra-provincial migrant workers is completely realized through the mediating role of social comparison, while the effect of income on that of inter-provincial ones is exerted through two ways: one is the direct effect of income itself, the other is through the indirect effect (mediating role) of social comparison. (3) Perceived discrimination plays a reverse moderating role in the process of social comparison affecting psychological integration: the lower the degree of perceived discrimination, the stronger the positive effect of social comparison on psychological integration, and vice versa. Therefore, according to the actual needs of different groups, relevant policies should be adjusted step-by-step to promote the psychological integration of migrant workers in China, thus improving their mental health level.

In addition, although China is still in the stage of large-scale urbanization, with the rapid advancement of China’s urbanization progress, the phenomenon of counter-urbanization or gentrification is gradually emerging. A large number of people are concentrated in cities, resulting in increasingly serious urban problems such as traffic congestion, environmental pollution, high housing prices, and the shortage of educational resources. As a result, the counter-urbanization phenomenon of urban population returning to the suburbs or rural areas appears in economically developed regions such as Guangdong, Jiangsu, Zhejiang, and Beijing. Otherwise, the residence, employment, and consumption of urban residents in the eastern coastal areas such as the Pearl River Delta and Yangtze River Delta, which have a high level of urbanization, began to expand to rural areas, which is similar to the phenomenon of rural gentrification. In China, these kinds of phenomenon have attracted increasing attention from policymakers and scholars. Therefore, research on related issues needs to be further developed.

## Figures and Tables

**Table 1:** Descriptive statistics of variables (sample size = 13,925).

Categorical Variables		Sample Size	Frequency	Continuous Variables	Mean	S.D.
Gender	Male	7639	54.9%	Age (years)	32.53	8.79
	Female	6286	45.1%	Average income of family (yuan/month)	6200	6400
Education	Primary or below	1444	10.4%	Time of current migration (year)	4.17	4.40
	Middle school	7695	55.3%	Working hours per week (hours)	55.89	20.43
	High school	3446	24.7%	Self-assessment of physical health (1–5)	3.76	0.97
	College	1070	7.7%	Mediating variable		
	Bachelor or above	270	1.9%	Social comparison (1–10)	5.45	1.59
Range of migration	Inter-provincial	7455	53.5%	Moderating variable		
	Intra-provincial	6470	46.5%	Perceived discrimination (1–4)	1.82	0.68
Free training	Accepted	4071	29.2%	Dependent variable		
	Have not accepted	9854	70.8%	Psychological belonging (1–4)	3.33	0.62

Data Source: The authors collated data from Special Survey on Social Integration and Mental Health of 2014 China Migrants Dynamic Survey National.

**Table 2:** Moderated mediating effect estimated results (Bootstrap = 5000).

Effects	Moderating Variable	Model 1					Model 2				
		Coefficient		S.E.	Bias-Corrected 95% CI		Coefficient		S.E.	Bias-Corrected 95% CI	
					Lower Bound	Upper Bound				Lower Bound	Upper Bound
Total effect		0.022	***	0.009	0.004	0.039	0.047	***	0.009	0.029	0.064
Direct effect		0.000		0.009	−0.017	0.017	0.021	*	0.009	0.004	0.039
Conditional indirect effect	Low value	0.023	***	0.003	0.018	0.028	0.022	***	0.003	0.017	0.028
	Median value	0.019	***	0.002	0.015	0.023	0.019	***	0.002	0.016	0.023
	High value	0.015	***	0.003	0.011	0.020	0.016	***	0.003	0.011	0.021

Note: The median of the moderating variable means that perceived discrimination is at the mean level. The high or low value is obtained by adding or subtracting one standard deviation from the mean, which means that perceived discrimination is at a high or low level. *** and * indicate significance at levels of 0.1% and 5%, respectively.

**Table 3:** Path estimation results of full-sample and inter-provincial migration model (Bootstrap = 5000).

Variable Categories	Variables	Social Comparison (Mediation Variable)						Psychological Belonging (Dependent Variable)					
		Model 1			Model 2			Model 1			Model 2		
		Coefficient		S.E.	Coefficient		S.E.	Coefficient		S.E.	Coefficient		S.E.
Intercept term		−3.998	***	0.181	−4.007	***	0.181	3.086	***	0.078	3.032	***	0.077
Control variables	Self-assessment of physical health	−0.254	***	0.012	−0.254	***	0.012	−0.052	***	0.005	−0.054	***	0.005
	Time of current migration	−0.002		0.003	−0.002		0.003	0.008	***	0.001	0.008	***	0.001
	Working hours per week	0.000		0.001	0.000		0.001	−0.001	*	0.000	−0.001	**	0.000
	Age	0.006	***	0.002	0.006	***	0.002	0.004	***	0.001	0.003	***	0.001
	Gender (Control group = Female)												
	Male	0.029		0.023	0.029		0.023	−0.017		0.010	−0.017		0.009
	Education (Control group = Primary or below)												
	Middle school	0.156	***	0.041	0.154	***	0.041	0.075	***	0.017	0.067	***	0.017
	High school	0.159	***	0.045	0.156	***	0.045	0.115	***	0.019	0.099	***	0.019
	College	0.118	*	0.053	0.114	*	0.053	0.121	***	0.023	0.098	***	0.022
	Bachelor or above	0.333	***	0.065	0.332	***	0.065	0.153	***	0.027	0.151	***	0.027
	Free training (Control group = Not accepted)												
	Accepted in past three years	0.147	***	0.024	0.141	***	0.025	0.136	***	0.010	0.102	***	0.010
Grouping variable	Range of migration (Control group = Intra-provincial)												
	Inter-provincial				−0.027		0.023				−0.164	***	0.010
Independent variable	Average monthly income of family	0.485	***	0.021	0.488	***	0.021	0.000		0.009	0.021	*	0.009
Mediating variable	Social comparison							0.039	***	0.003	0.039	***	0.003
Moderating variable	Perceived discrimination							−0.209	***	0.007	−0.196	***	0.007
Interaction term	Social comparison × Perceived discrimination							−0.011	**	0.004	−0.010	*	0.004

Note: The Bootstrap 95% confidence interval (CI) method two-tailed test is used. The upper and lower bounds of CI are not listed due to space reasons. ***, **, and * indicate significance at levels of 0.1%, 1% and 5%, respectively.

## Data Availability

The original data of this study was obtained by submitting a formal application to Migrant Population service Center, National Health Commission P.R. China on the website www.chinaldrk.org.cn.

## References

[1] National Bureau of Statistics Statistical Bulletin of National Economic and Social Development of the People’s Republic of China in 2020. http://www.stats.gov.cn/tjsj/zxfb/202102/t20210227_1814154.html.

[2] Chen Y., Zhang Y., Guozhen Z. (2015). The inequality effect and social integration in urbanization. Soc. Sci. China.

[3] Yang L. (2018). Research on the Heterogeneity of Migrant Workers Group from the Perspective of Precision Governance. Study Pract..

[4] Yang J. (2009). Segregation, Selective Assimilation and Assimilation: A Conceptual Framework of Rural to Urban Migrant’s adaptation at destination. Popul. Res..

[5] Zhang W., Lei K. (2008). The urban new immigrants’ social inclusion: Internal structure, present situation and influential factors. Sociol. Stud..

[6] Yang J. (2019). Citizenization of migrants: Theory, reality and reflection. Jilin Univ. J. Soc. Sci. Ed..

[7] Cui Y. (2012). A study on migrants’ psychological integration and self-identity. Sociol. Stud..

[8] Lewis W.A. (1954). Economic development with unlimited supplies of labor. Manch. Sch. Econ. Soc. Stud..

[9] Bouge D.J., Hauser P.M., Duncan O.D. (1959). Internal Migration. The Study of Population.

[10] Schultz T.W. (1960). Capital formation by education. J. Political Econ..

[11] Harris J.R., Todaro M.P. (1970). Migration, unemployment and development: A two-sector analysis. Am. Econ. Rev..

[12] Berry B.J.L. (1976). Urbanization and Counterurbanization.

[13] Cloke P. (1985). Counterurbanisation: A rural perspective. Geography.

[14] Phillips M. (1993). Rural gentrification and the processes of class colonisation. J. Rural Stud..

[15] Phillips M. (2010). Counterurbanisation and rural gentrification: An exploration of the terms. Popul. Space Place.

[16] Gordon M.M. (1964). Assimilation in American Life: The Role of Race, Religion, and National Origins.

[17] Phinney J.S. (1990). Ethnic identity in adolescents and adults: Review of research. Psychol. Bull..

[18] Phinney J.S., Horenczyk G., Liebkind K., Vedder P. (2001). Ethnic identity, immigration, and well-being: An interactional perspective. J. Soc. Issues.

[19] Yue Z.S., Li S.Z., Feldman M.W. (2012). Concept construction and empirical analysis of social integration for rural-urban migrants in China. Mod. Econ. Sci..

[20] Yang J. (2015). Research on the assimilation of the floating population in China. Soc. Sci. China.

[21] Li T.C., Chu C.C., Meng F.C., Li Q., Mo D., Li B., Tsai S.-B. (2018). Will happiness improve the psychological integration of migrant workers?. Int. J. Environ. Res. Public Health.

[22] Zhang Y., Cen Q. (2014). Spatial patterns of population mobility and determinants of inter-provincial migration in China. Popul. Res..

[23] Yang C., Li W., Lu Y. (2014). Impact of migrant workers income and working hours on their life satisfaction: Urban integration and the role of the social security. J. Agrotech. Econ..

[24] Meng Y., Deng D. (2011). The “income paradox” in the course of migrant workers integrating into cities: The case of Wuhan city. Chin. J. Popul. Sci..

[25] Gilbert D.T., Giesler R.B., Morris K.A. (1995). When comparisons arise. J. Personal. Soc. Psychol..

[26] Festinger L. (1954). A Theory of Social Comparison Processes. Hum. Relat..

[27] Wang J.L., Wang H.Z., Gaskin J., Hawk S. (2017). The mediating roles of upward social comparison and self-esteem and the moderating role of social comparison orientation in the association between social networking site usage and subjective well-being. Front. Psychol..

[28] Park S.Y., Baek Y.M. (2018). Two faces of social comparison on Facebook: The interplay between social comparison orientation, emotions, and psychological well-being. Comput. Hum. Behav..

[29] Cobb C.L., Meca A., Branscombe N.R., Schwartz S.J., Xie D., Zea M.C., Fernandez C.A., Sanders G.L. (2019). Perceived discrimination and well-being among unauthorized Hispanic immigrants: The moderating role of ethnic/racial group identity centrality. Cult. Divers. Ethn. Minority Psychol..

[30] Tartakovsky E., Patrakov E., Nikulina M. (2021). Is emigration worth the trouble? Satisfaction with life, group identifications, perceived discrimination, and socio-economic status of immigrants and stayers. Int. J. Intercult. Relat..

[31] Szaflarski M., Bauldry S. (2019). The Effects of Perceived Discrimination on Immigrant and Refugee Physical and Mental Health. Immigration and Health (Advances in Medical Sociology, Volume 19).

[32] Song Y., Tao Y. (2012). Assimilation and acceptance: An empirical study on migrants’ social assimilation from interactive perspective. Popul. Res..

[33] Fuller-Rowell T.E., Ong A.D., Phinney J.S. (2013). National identity and perceived discrimination predict changes in ethnic identity commitment: Evidence from a longitudinal study of Latino College students. Appl. Psychol..

[34] Zhang L., Zhang Y. (2015). Review of social isolation in the vulnerable groups. J. Chang. Univ. Sci. Technol..

[35] Yuksek D.A., Solakoglu O. (2016). The relative influence of parental attachment, peer attachment, school attachment, and school alienation on delinquency among high school students in Turkey. Deviant Behav..

[36] Ifeagwazi C.M., Chukwuorji J.B.C., Zacchaeus E.A. (2015). Alienation and psychological wellbeing: Moderation by resilience. Soc. Indic. Res..

[37] Schmitt M.T., Branscombe N.R., Postmes T., Garcia A. (2014). The consequences of perceived discrimination for psychological well-being: A meta-analytic review. Psychol. Bull..

[38] Berry J.W., Azzi A.E., Chryssochoou X., Klandermans B., Simon B. (2010). Immigrant acculturation: Psychological and social adaptations. Identity and Participation in Culturally Diverse Societies: A Multidisciplinary Perspective.

[39] Hayes A.F. (2017). Introduction to Mediation, Moderation, and Conditional Process Analysis: A Regression-Based Approach.

[40] Hayes A.F., Rockwood N.J. (2020). Conditional Process Analysis: Concepts, Computation, and Advances in the Modeling of the Contingencies of Mechanisms. Am. Behav. Sci..

[41] Hoggart K., Buller H. (1987). Rural Development: A Geographical Perspective.

[42] Halfacree K.H. (1994). The importance of “the rural” in the constitution of counterurbanization: Evidence from England in the 1980s. Sociol. Rural..

[43] Boyle P., Halfacree K. (1998). Migration into Rural Areas: Theories and Issues.

[44] Hoggart K., Mendoza C. (1999). African immigrant workers in Spanish agriculture. Sociol. Rural..

[45] Halfacree K. (2001). Constructing the object: Taxonomic practices, ‘counterurbanization’ and positioning marginal rural settlement. Int. J. Popul. Geogr..

[46] Paniagua Á. (2002). Urban-rural migration, tourism entrepreneurs and rural restructuring in Spain. Tour. Geogr..

[47] Champion T., Hugo G. (2004). New Forms of Urbanization: Beyond the Urban-Rural Dichotomy.

[48] Halfacree K. (2011). Radical spaces of rural gentrification. Plan. Theory Pract..

[49] Halfacree K. (2012). Heterolocal identities? Counter-urbanisation, second homes, and rural consumption in the era of mobilities. Popul. Space Place.

[50] Phillips M., Smith D.P. (2018). Comparative approaches to gentrification: Lessons from the rural. Dialogues Hum. Geogr..

